# Supplementary material for: Hedgehog Signalling Modulates Immune Response and Protects against Experimental Autoimmune Encephalomyelitis
Source: Int J Mol Sci. 2022 Mar 15;23(6):3171. doi: 10.3390/ijms23063171 (PMC8954986; doi:10.3390/ijms23063171)
Supplement: Supplementary file 1 [file ijms-23-03171-s001.zip › ijms-1625312-supplementary.pdf]

Figure S1

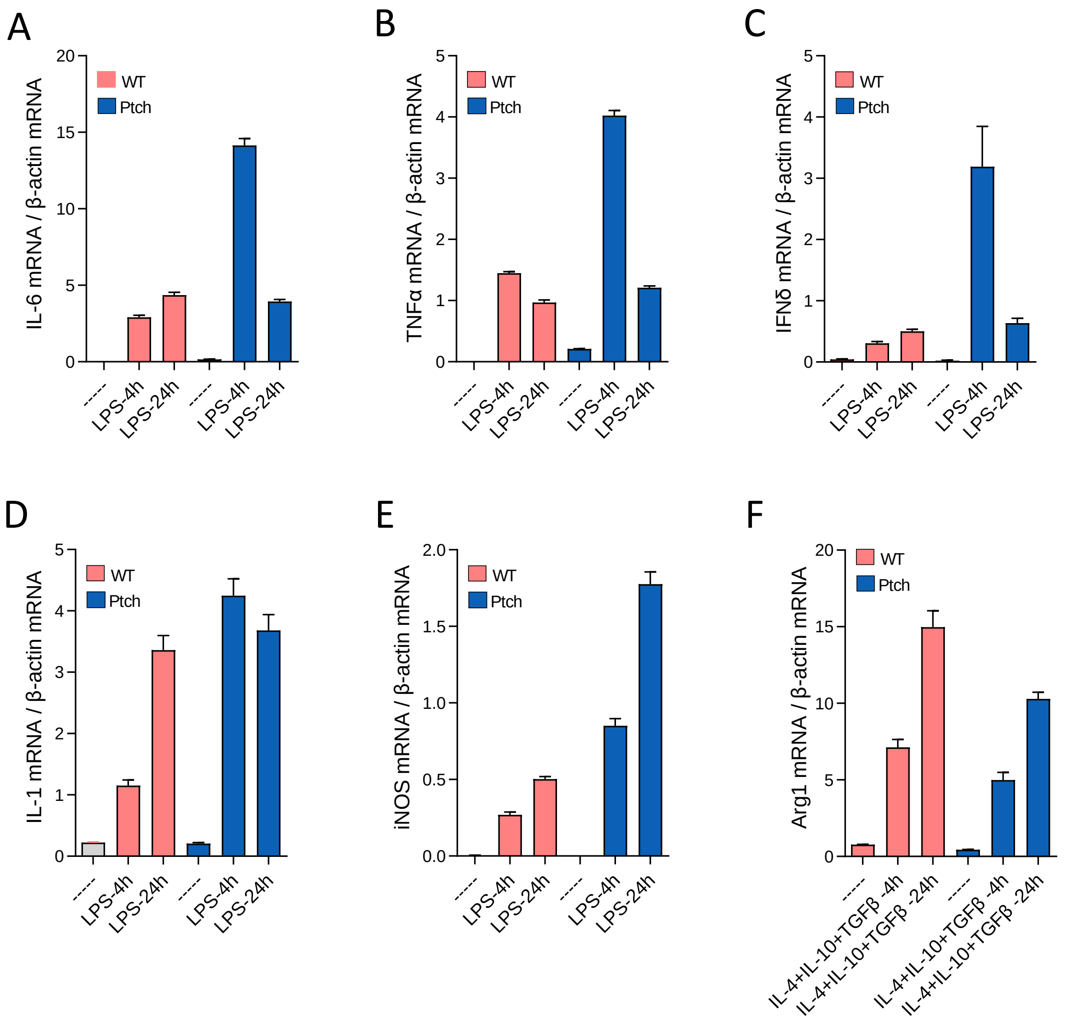

Supplementary Figure S1: Kinetics of gene induction in macrophages from *ptch*$^{+/-}$ and wt mice. Peritoneal macrophages were cultured in M1 (A-E) or M2 (F) conditions. At 4 or 24 hours under stimulation, specified for each gene, cells were subjected to RNA extraction and the gene expression of the products indicated in each panel were quantified by RT-PCR. Data are the mean of three independent experiments in which each sample was assayed in triplicate.

Figure S2

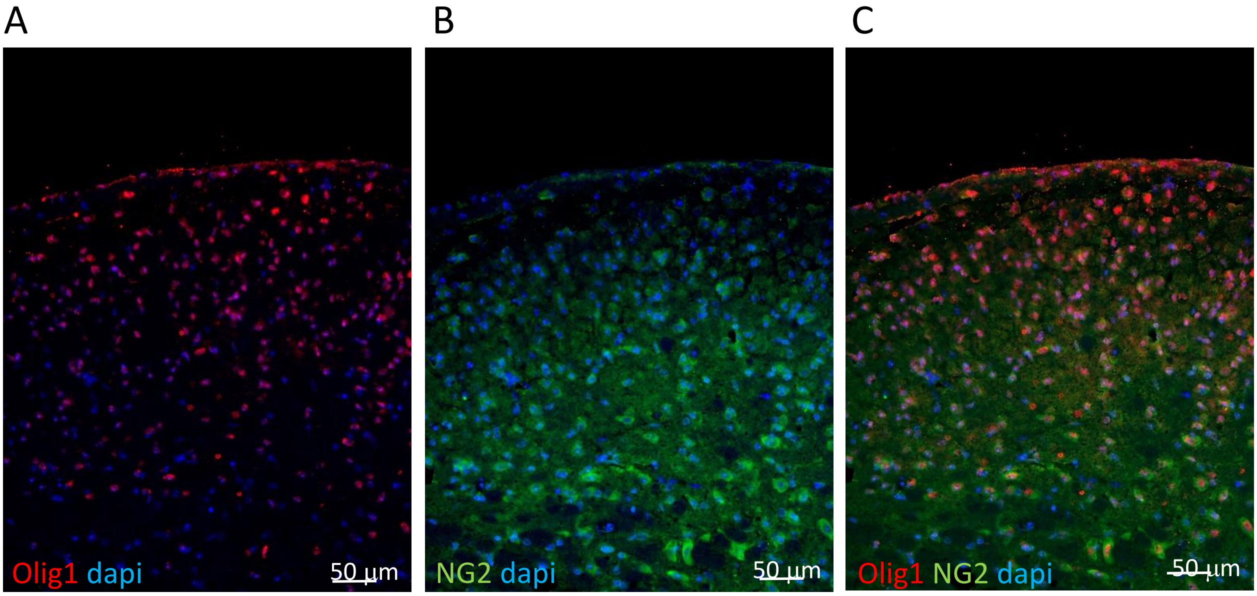

Supplementary Figure S2: Immunofluorescence performed with anti-Olig1 (A), anti-NG2 (B) antibodies, or double staining (C) in spinal cord section from a *ptch*$^{+/-}$ mouse. Nuclei in all micrographs show DAPI staining (blue). All NG2 positive cells are also positive for Olig1.

Figure S3

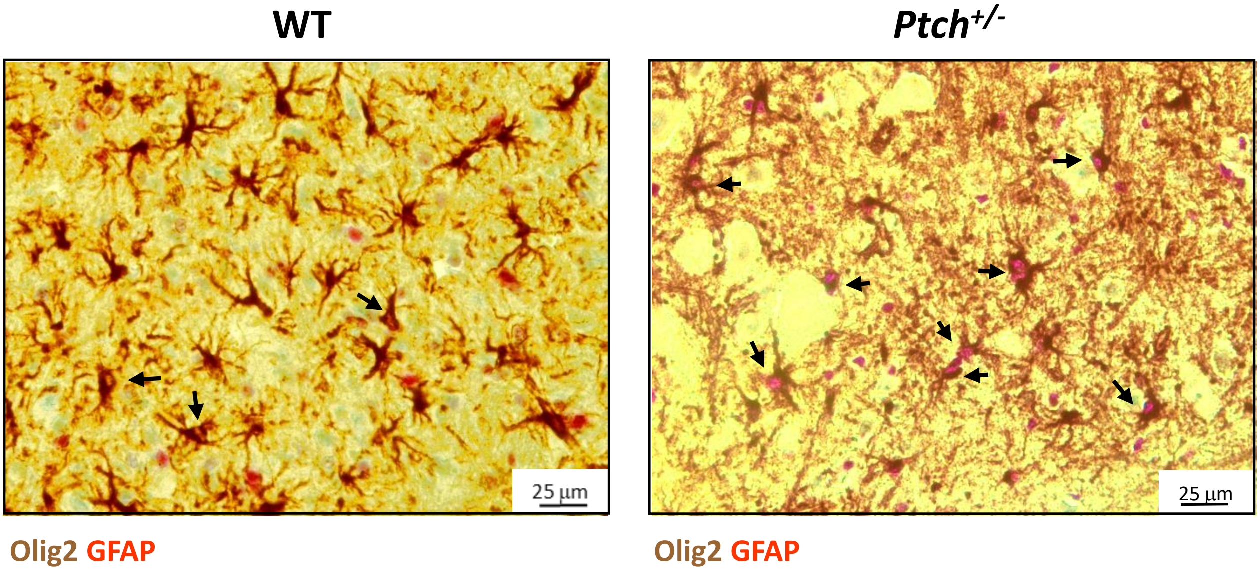

Supplementary Figure S3: Mice with same levels of response to EAE induction were selected from wt and *ptch* $^{(+/-)}$ groups after MOG_{35-55} inoculation. Double immunohistochemistry performed with anti-Olig2 (red) and anti-GFAP (brown) antibodies on spinal cord sections revealing higher number of Olig2/GFAP double positive cells in *ptch*$^{+/-}$ than in wt sections (arrows).

Figure S4

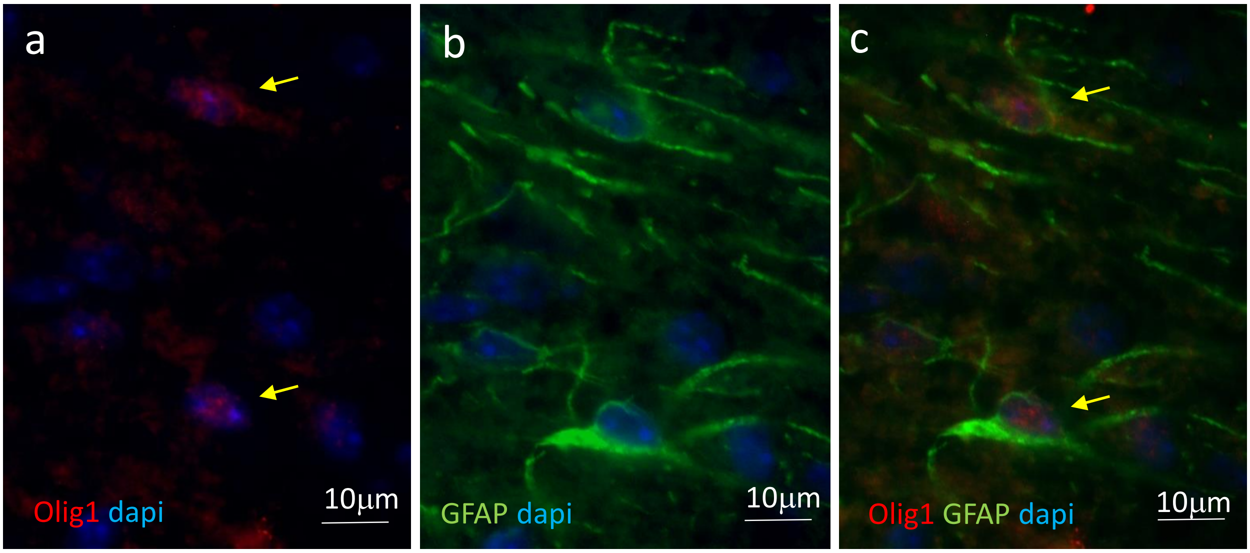

Supplementary Figure S4: Immunofluorescence with anti-Olig1 (a) and anti-GFAP (b) antibodies, or double staining (c) on neural tissue sections from a *ptch*$^{+/-}$ mice inoculated with $MOG_{35\text{-}55}$ and sacrificed at day 20 post-inoculation. Olig1^{+} nuclei and GFAP^{+} cytoplasms are dyed red and green, respectively. Nuclei in all micrographs show DAPI staining (blue), yellow arrows show Olig1^{+}GFAP^{+} cells.

Figure S5

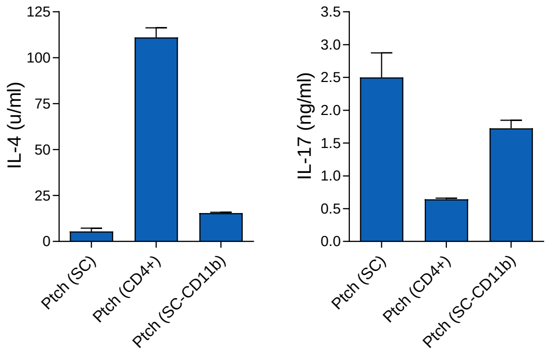

Supplementary Figure S5: IL-4 and IL-17 production in spleen cells from *ptch*$^{+/-}$. Total, CD11b^{+}-depleted or purified CD4^{+} cells from spleen of *ptch+/-* mice (10^{6}/ml), were stimulated with aCD3/aCD28. The production of IL-4 and IL-17 was quantified by ELISA after three days of culture.

Figure S6

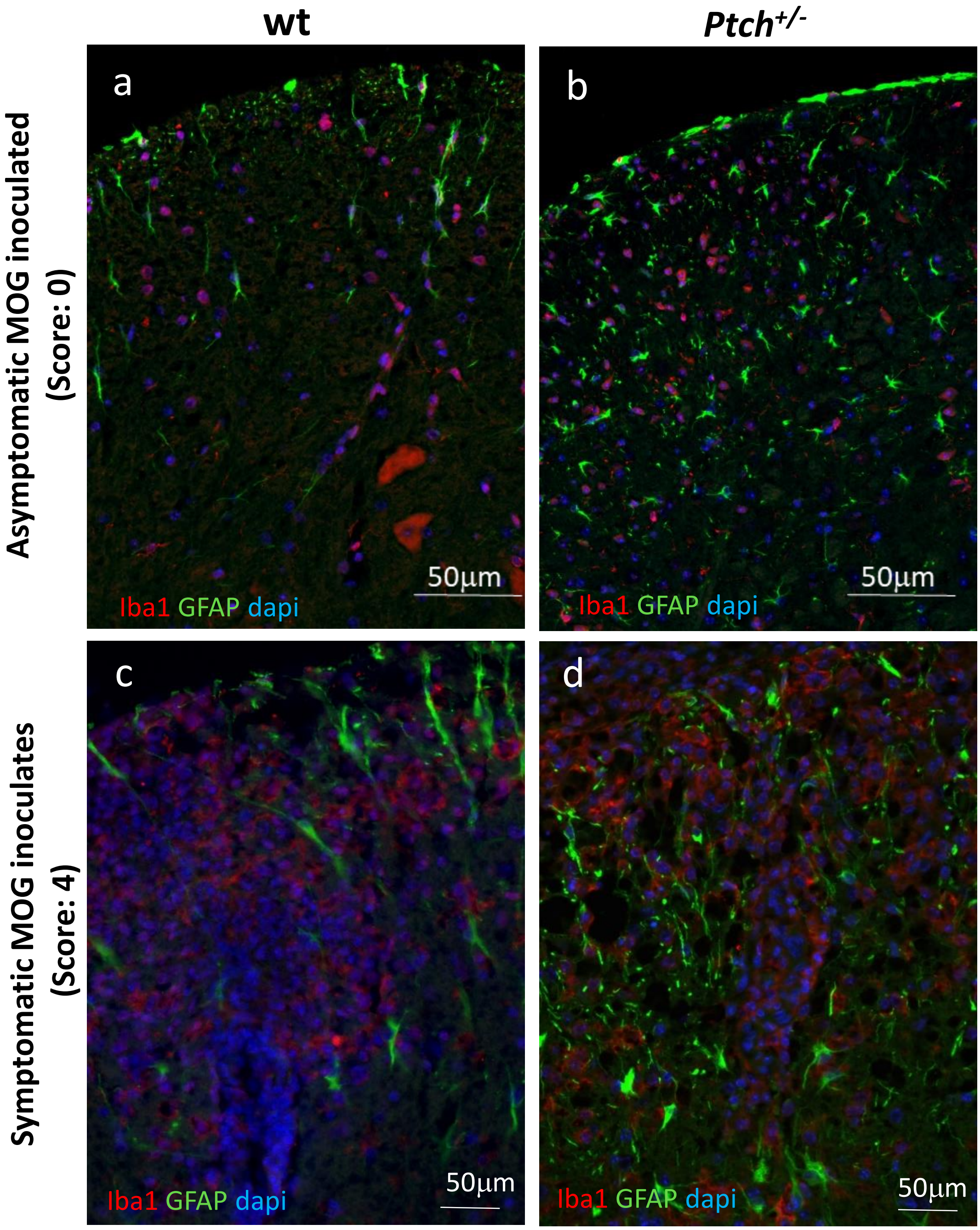

Supplementary Figure S6: Mice with different levels of response to EAE induction were selected from wt (a and c) and *ptch*$^{+/-}$ (b and d) groups after $MOG_{35\text{-}55}$ inoculation. Double Immunofluorescence with anti-GFAP/anti-Iba1 antibodies showing the outer part of spinal cord cross sections. Both Iba1 (red) and GFAP (green) expression is higher in EAE with high score (c and d) than in asymptomatic (a and b) spinal cords, but while GFAP shows sparse but thick staining marks in wt mice, GFAP^{+} cells are smaller but more numerous in *ptch*$^{+/-}$mice both symptomatic or asymptomatic. In all four slides nuclei where stained by DAPI (blue).
